# Twist1 Controls Lung Vascular Permeability and Endotoxin-Induced Pulmonary Edema by Altering Tie2 Expression 

**DOI:** 10.1371/journal.pone.0073407

**Published:** 2013-09-02

**Authors:** Tadanori Mammoto, Elisabeth Jiang, Amanda Jiang, Yongbo Lu, Aimee M. Juan, Jing Chen, Akiko Mammoto

**Affiliations:** 1 Vascular Biology Program, Department of Surgery, Boston Children's Hospital and Harvard Medical School, Boston, Massachusetts, United States of America; 2 Department of Biomedical Sciences, Baylor College of Dentistry, Texas A&M Health Science Center, Dallas, Texas, United States of America; 3 Department of Ophthalmology, Boston Children's Hospital and Harvard Medical School, Boston, Massachusetts, United States of America; University of Illinois College of Medicine, United States of America

## Abstract

Tight regulation of vascular permeability is necessary for normal development and deregulated vascular barrier function contributes to the pathogenesis of various diseases, including acute respiratory distress syndrome, cancer and inflammation. The angiopoietin (Ang)-Tie2 pathway is known to control vascular permeability. However, the mechanism by which the expression of Tie2 is regulated to control vascular permeability has not been fully elucidated. Here we show that transcription factor Twist1 modulates pulmonary vascular leakage by altering the expression of Tie2 in a context-dependent way. Twist1 knockdown in cultured human lung microvascular endothelial cells decreases Tie2 expression and phosphorylation and increases RhoA activity, which disrupts cell-cell junctional integrity and increases vascular permeability *in vitro*. In physiological conditions, where Ang1 is dominant, pulmonary vascular permeability is elevated in the Tie2-specific Twist1 knockout mice. However, depletion of Twist1 and resultant suppression of Tie2 expression prevent increase in vascular permeability in an endotoxin-induced lung injury model, where the balance of Angs shifts toward Ang2. These results suggest that Twist1-Tie2-Angs signaling is important for controlling vascular permeability and modulation of this mechanism may lead to the development of new therapeutic approaches for pulmonary edema and other diseases caused by abnormal vascular permeability.

## Introduction

Tightly regulated vascular permeability is indispensable for organ development and function [1], while compromised vascular barrier function contributes to many pathological conditions, including acute respiratory distress syndrome (ARDS), atherosclerosis, cancer, and organ failure [1–7]. A number of soluble inflammatory regulators such as vascular endothelial growth factor (VEGF) [4,8], tumor necrosis factor (TNF)-α [9], transforming growth factor (TGF)-β [10], and IL2 [11] as well as their receptors are known to regulate endothelial barrier function. In addition, recently, we and other groups have reported that the angiopoietin (Ang) -Tie2 pathway mediates endotoxin-induced lung injury [3,12,13] and bronchopulmonary dysplasia (BPD) [14], a neonatal lung injury accompanied by an increase in lung vascular permeability [15,16]. However, the precise mechanism by which Tie2 expression is controlled and regulates vascular permeability remains to be elucidated.

Twist1, a transcription factor identified in mouse by its high similarity to Drosophila Twist [17], controls mammalian embryonic development, including limb budding and cranial neural tube closure [18–21]. Mouse embryos homozygous for the Twist1-targeted mutation die at E11.5 because of failure of cranial neural tube closure [21,22]. Twist1 also contributes to normal and tumor angiogenesis [23–26] as well as the epithelial-mesenchymal transition in lung fibrosis [27] and lung cancer [28], where vascular permeability is increased. Importantly, Twist1 has a b-HLH sequence that binds to a consensus sequence called an E-box (CANNTG) [18,29], which is present in the promoter region of Tie2 [30]. Given that the Ang-Tie2 pathway mediates endotoxin-induced lung injury [3,12] and that Tie2 has an E-box region in its promoter region [30], Twist1 may control lung vascular barrier function by modulating the expression of Tie2.

ARDS is a life-threatening respiratory complication that often accompanies sepsis. In ARDS, increased lung vascular permeability causes pulmonary edema [31,32], which impairs gas exchange across the alveolar membrane and severely compromises respiratory function. ARDS occurs in almost half of human patients with severe sepsis [31], and the mortality rate of sepsis-induced ARDS is higher than 60% [33]. Despite a large amount of effort to develop specific clinical therapies for ARDS, currently there is no efficient therapy for this devastating disease or any other conditions accompanied by abnormal vascular permeability.

In this study, we show that Twist1 knockdown disrupts cell-cell junctional integrity and increases vascular permeability by suppressing Tie2 expression *in vitro* and *in vivo*. We also show that downregulation of Twist1-Tie2 signaling prevents increase of lung vascular permeability and restores lung function in a mouse endotoxin-induced lung injury model. The Twist1-Tie2 pathway might therefore represent a new target for therapeutic strategies for sepsis-induced ARDS.

## Results

### Twist1 knockdown decreases Tie2 expression in vitro

**Figure 1:**
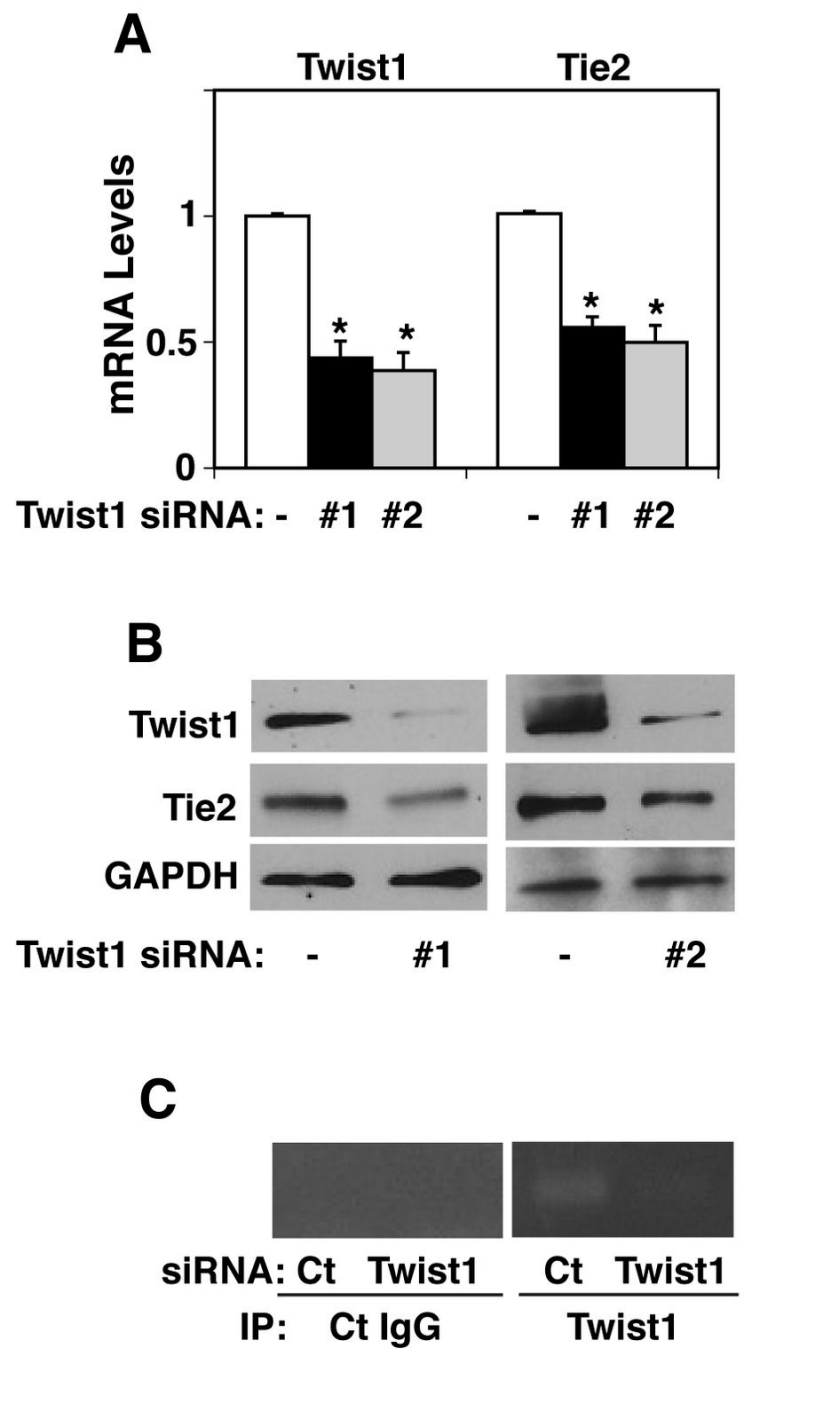
Twist1 controls Tie2 expression in microvascular endothelial cells. **A**) Graph showing Twist1 and Tie2 mRNA levels in L-HMVE cells treated with Twist1 siRNA #1 or #2 (* p<0.01). As a control, cells were treated with siRNA duplex with an irrelevant sequence. Error bars represent s.e.m. of at least three independent experiments. **B**) Immunoblots showing Twist1, Tie2, and GAPDH protein levels in L-HMVE cells treated with Twist1 siRNA #1 or #2. **C**) ChIP analysis showing Tie2 promoter co-immunoprecipitating (IP) with control IgG or Twist1 antibody in L-HMVE cells transfected with Twist1 siRNA #1. Ct, control.

Twist1 is a b-HLH transcription factor which binds to the E-box promoter region [18,29]. Since the Tie2 promoter has E-box consensus sequences [30], we first examined whether Twist1 controls Tie2 expression in lung human microvascular endothelial (L-HMVE) cells *in vitro*. Knockdown of Twist1 using siRNA (#1) transfection, which decreased Twist1 expression levels by half, downregulated mRNA levels of Tie2 in L-HMVE cells by half (Figure 1A). Knockdown of Twist1 also downregulated protein levels of Twist1 and Tie2 in L-HMVE cells when analyzed using immunoblotting (Figure 1B). Similar knockdown effects on Tie2 were obtained using a second Twist1 siRNA (#2), suggesting that the knockdown effect of Twist1 is not an off-target effect of siRNA (Figure 1A, B). Since Twist1 is a transcription factor, we next examined whether Twist1 binds the Tie2 promoter region in L-HMVE cells using chromatin immunoprecipitation (ChIP) assay [34] (Figure 1C). ChIP analysis showed that Twist1 binds the Tie2 promoter region (-666--455), which includes the E-box, and Twist1 knockdown using siRNA (#1) transfection resulted in decreased binding, indicating that Twist1 binds the Tie2 promoter. Control IgG did not immunoprecipitate these DNAs in L-HMVE cells (Figure 1C).

### Twist1 knockdown increases vascular permeability in vitro

**Figure 2:**
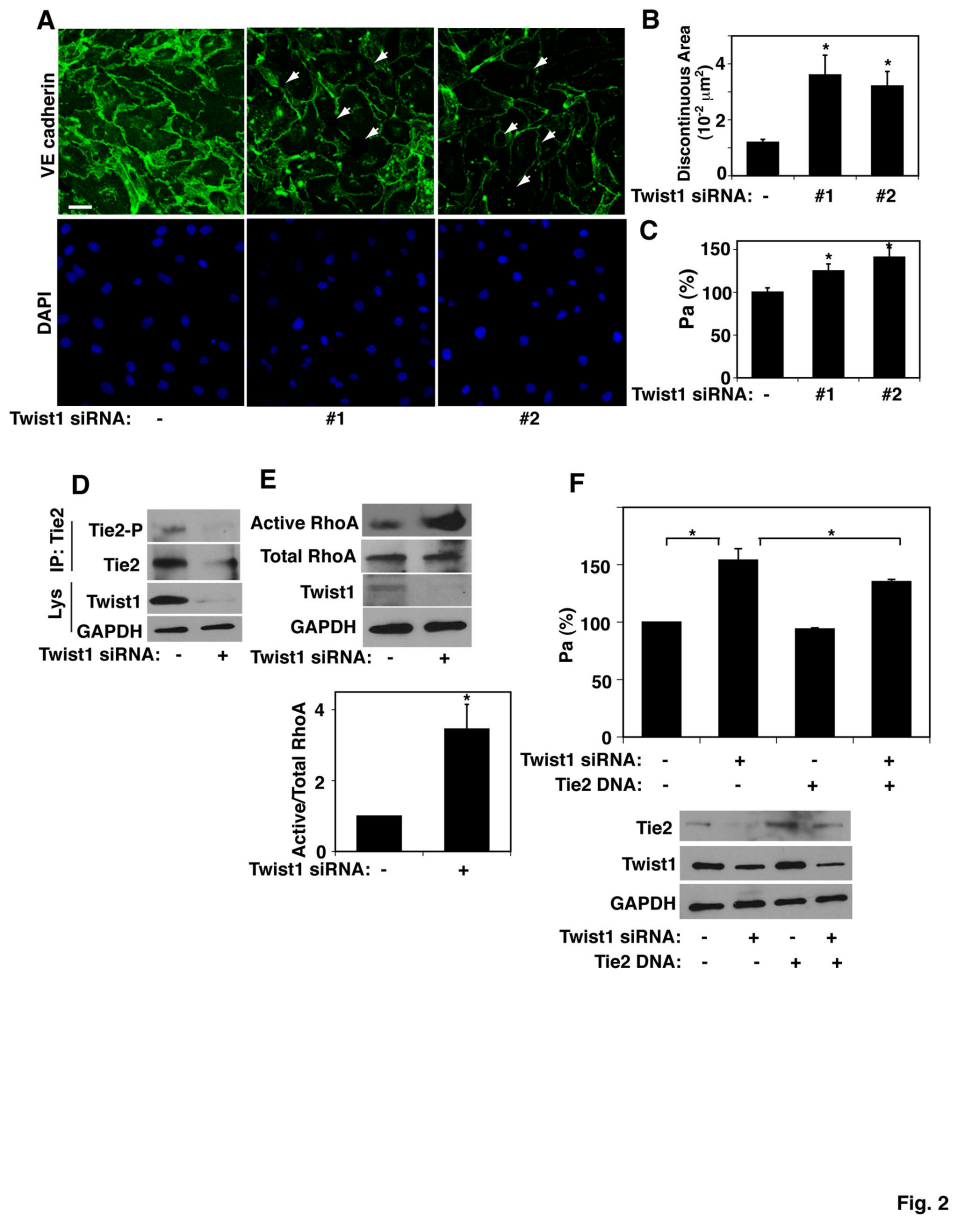
Twist1 controls the integrity of endothelial cell-cell junction in microvascular endothelial cells *in vitro*. **A**) Immunofluorescence micrographs showing cell-cell junction structure by VE-cadherin staining in L-HMVE cells treated with Twist1 siRNA #1 or #2 (bar, 50 µm). DAPI staining shows the nuclei of cells. Arrows show the region where cell-cell junctions are disrupted. As a control, cells were treated with siRNA duplex with an irrelevant sequence. **B**) Graph showing the quantitation of the total discontinuous area of at least 10 fields (*, p<0.01). **C**) Graph showing endothelial permeability in L-HMVE cells treated with control siRNA, Twist1 siRNA #1 or #2 (*, p<0.05). Permeability (Pa) values were evaluated after 6 h and are expressed as percentage of control cells. **D**) Immunoblots showing tyrosine phosphorylated Tie2 detected by phosphotyrosine antibody (4G10) and total Tie2 immunoprecipitated with Tie2 antibody from total cell lysates in Twist1 knockdown (siRNA #1 treated) L-HMVE cells. **E**) Levels of active RhoA in Twist1 knockdown (siRNA #1 treated) L-HMVE cells. Quantitative results (ratio of active RhoA to total RhoA) were normalized to control siRNA treated cells (*, p<0.01). **F**) Graph showing endothelial permeability in HUVE cells treated with Twist1 siRNA #1, Tie2 DNA, or in combination (*, p<0.05). As a control, cells were treated with siRNA duplex with an irrelevant sequence and/or control DNA (vector only). Immunoblots showing Twist1, Tie2, and GAPDH protein levels in HUVE cells treated with Twist1 siRNA #1, Tie2 DNA, or in combination. All error bars are s.e.m.

Since Tie2 is known to control vascular permeability [3,12] and our *in vitro* results indicate that Twist1 controls Tie2 expression (Figure 1), we next explored the possibility that Twist1 might be involved in control of vascular barrier function. VE-cadherin-containing cell-cell junctions resist the traction forces generated in the contractile actin cytoskeleton in cells, and hence control vascular permeability [3]. Immunocytochemical analysis revealed that normally well-defined, linear cell-cell junctions were disrupted when Twist1 mRNA and protein levels were knocked down in cultured L-HMVE cells using two distinct siRNAs (#1 and #2) (Figures 1 and 2A). Quantitative results revealed that the discontinuous area was increased by 3 times in Twist1 knockdown cells compared to L-HMVE cells treated with control siRNA with irrelevant sequence (Figure 2B). Twist1 knockdown also increased vascular permeability by 1.3-fold as measured by quantitating the flux of fluorescently-labeled albumin across the cell monolayer cultured in a Transwell chamber *in vitro* (Figure 2C) [3]. These knockdown effects were obtained by two distinct siRNAs against Twist1, suggesting that these knockdown effects of Twist1 siRNA on cell-cell junction integrity are not off-target effects of siRNA (Figure 2A–C) and we used Twist1 siRNA #1 for the rest of the experiments. It has been reported that phosphorylation of the Tie2 receptor and subsequent change in Rho small GTPase activity control vascular permeability in both cultured endothelial cells *in vitro* and *in vivo* in lungs [3,12,35]. Thus, we next examined whether Twist1 controls Tie2 phosphorylation and RhoA activity in L-HMVE cells. Twist1 knockdown significantly decreased Tie2 expression as well as Tie2 phosphorylation in L-HMVE cells (Figure 2D). When we examined RhoA activity using a rhotekin pull down assay, RhoA activity was three times higher in Twist1-knocked down L-HMVE cells compared to cells treated with control siRNA with irrelevant sequence (Figure 2E). Importantly, when we over-expressed Tie2 in HUVE cells using DNA transfection, the Twist1 knock down-induced vascular leakage was partially inhibited (Figure 2F). These results indicate that Twist1 regulates vascular permeability by changing the expression levels and associated phosphorylation status of Tie2 and changing RhoA activity in microvascular endothelial cells.

### Twist1 knockdown increases vascular permeability in the mouse lung in vivo

**Figure 3:**
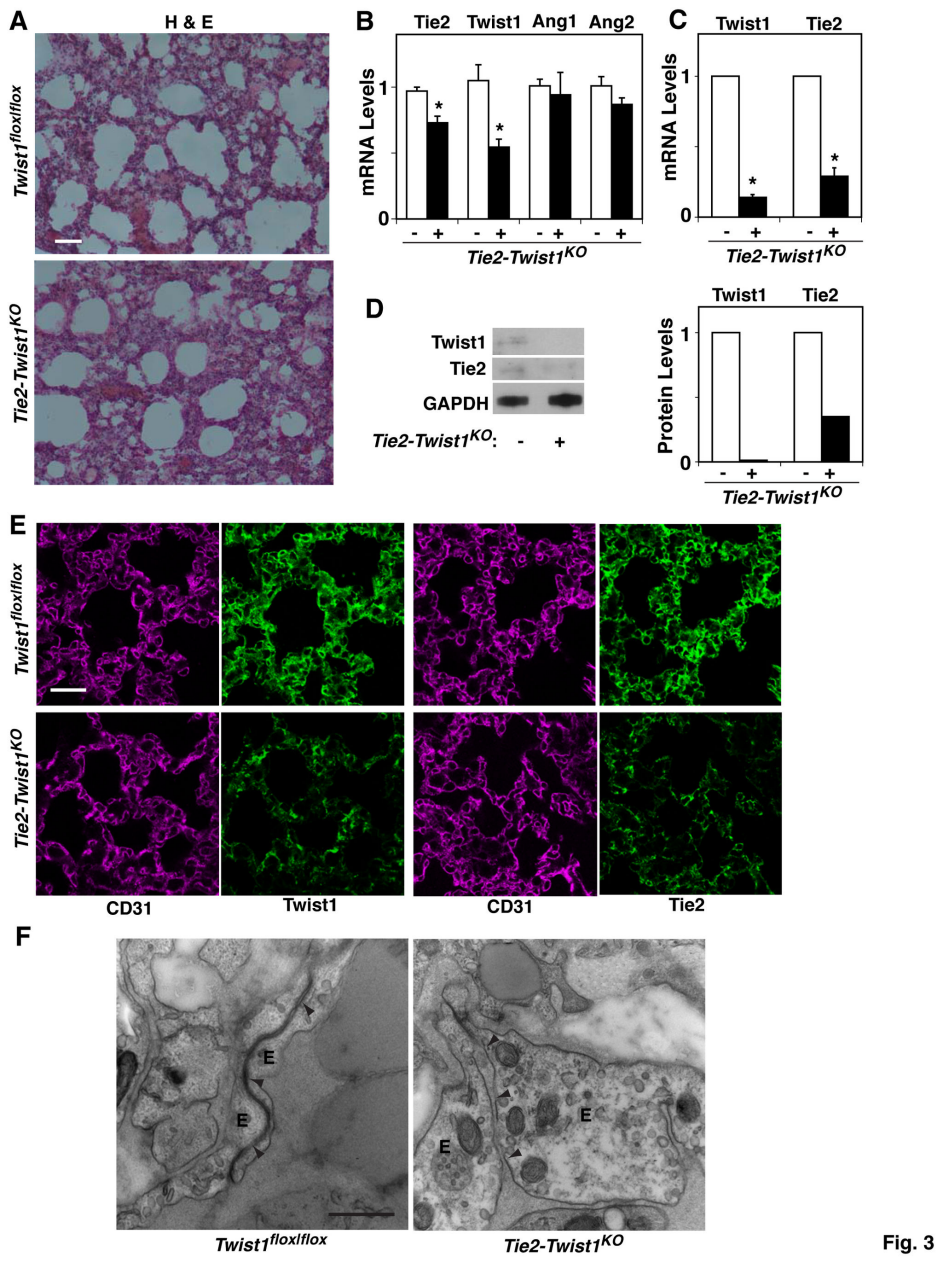
Twist1 regulates endothelial microstructure and cell-cell junctional integrity in the mouse lung *in vivo*. **A**) H&E staining of the lungs from control *Twist1* ^*flox|flox*^ or *Tie2-Twist1* ^KO^ mice. Scale bar, 50 µm. **B**) Graph showing Twist1, Tie2, and Ang1, 2 mRNA levels in the lungs of *Twist1* ^*flox|flox*^ or *Tie2-Twist1* ^KO^ mice (n=8, * p<0.05). Error bars are s.e.m. **C**) Graph showing Twist1 and Tie2 mRNA levels in the lung blood vessels of *Twist1* ^*flox|flox*^ or *Tie2-Twist1* ^KO^ mice isolated using LCM (* p<0.05). Error bars are s.e.m. **D**) Immunoblots showing Twist1, Tie2 and GAPDH protein levels in CD31 positive cells isolated from *Twist1* ^*flox|flox*^ or *Tie2-Twist1* ^KO^ mice (left). Graphs showing the quantitative results of protein levels of Twist1 and Tie2 normalized to GAPDH protein levels (right). **E**) Immunofluorescence micrographs showing expression and distribution of Twist1, Tie2 and CD31-stained blood vessels in the lungs of *Twist1* ^*flox|flox*^ or *Tie2-Twist1* ^KO^ mice (bar, 20 µm). **F**) TEM images showing endothelial microstructure and cell-cell junctional integrity in the lungs of *Twist1* ^*flox|flox*^ or *Tie2-Twist1* ^KO^ mice (bar, 500 nm). E: endothelial cells. Arrowheads show the region of cell-cell junctions.

To further evaluate the role of Twist1 in vascular permeability *in vivo*, we created Tie2-specific conditional Twist1 knockout mice (*Tie2-Twist1* ^*KO*^) by crossing Tie2-Cre expressing mice with *Twist1* ^*flox|flox*^ mice exhibiting floxed disruption in the *Twist1* gene. Histology (H & E staining) of lung sections revealed that the interstitial wall is thicker throughout the lungs of *Tie2-Twist1* ^KO^ mice (Figure 3A, 6 weeks old) compared to the lungs of age matched control *Twist1* ^*flox|flox*^ mice. Twist1 and Tie2 mRNA expression decreased by 50% and 20% respectively in the whole lungs of *Tie2-Twist1* ^KO^ mice (Figure 3B), which is consistent with the *in vitro* data showing that knockdown of Twist1 decreases Tie2 expression (Figure 1). The expression of the Tie2 ligands, Ang1 and 2, was not altered in the lungs of *Tie2-Twist1* ^*KO*^ mice compared to that in control *Twist1* ^*flox|flox*^ mice (Figure 3B). We also examined the degree of Twist1 and Tie2 inhibition in endothelial cells of *Tie2-Twist1* ^*KO*^ mouse lungs using laser capture microdissection (LCM) on unfixed frozen sections of lung tissue. We collected concanavalin A labeled endothelial cells and measured mRNA levels of Twist1 and Tie2 in endothelial cells using qRT-PCR. Twist1 and Tie2 expression were lower by 90% and 75%, respectively in lung endothelial cells from *Tie2-Twist1* ^*KO*^ mice compared to that in control *Twist1* ^*flox|flox*^ mice (Figure 3C). We also enzymatically digested lungs of these mice and isolated endothelial cells by incubating these cells with CD31-coated beads and analyzed the protein levels of Twist1 and Tie2 in the cells using immunoblotting. Consistent with the results obtained by LCM, Twist1 and Tie2 expression were lower by 98% and 65%, respectively in lung endothelial cells collected from *Tie2-Twist1* ^*KO*^ mice compared to cells from control *Twist1* ^*flox|flox*^ mice (Figure 3D). We further confirmed the results using immunohistochemical analysis, showing that Twist1 and Tie2 expression were lower in CD31-positive endothelial cells of *Tie2-Twist1* ^*KO*^ mouse compared to those of control *Twist1* ^*flox|flox*^ mouse (Figure 3E). The junctional integrity and endothelial microstructure of the lungs were also analyzed in control *Twist1* ^*flox|flox*^ and *Tie2-Twist1* ^KO^ mouse lungs using transmission electron microscopy (TEM). Junctions between pulmonary endothelial cells were tight and characterized by closely apposed membranes in randomly sampled regions of lungs from control *Twist1* ^*flox|flox*^ mice. In contrast, in lungs from *Tie2-Twist1* ^KO^ mice, endothelial cells were swollen with cubical shape and cell-cell junctions appeared to be disrupted with increased space appearing between adjacent cell membranes (Figure 3F). These findings support the hypothesis that Twist1-Tie2 signaling plays a key role in endothelial cell structure and junctional integrity *in vivo*.

**Figure 4:**
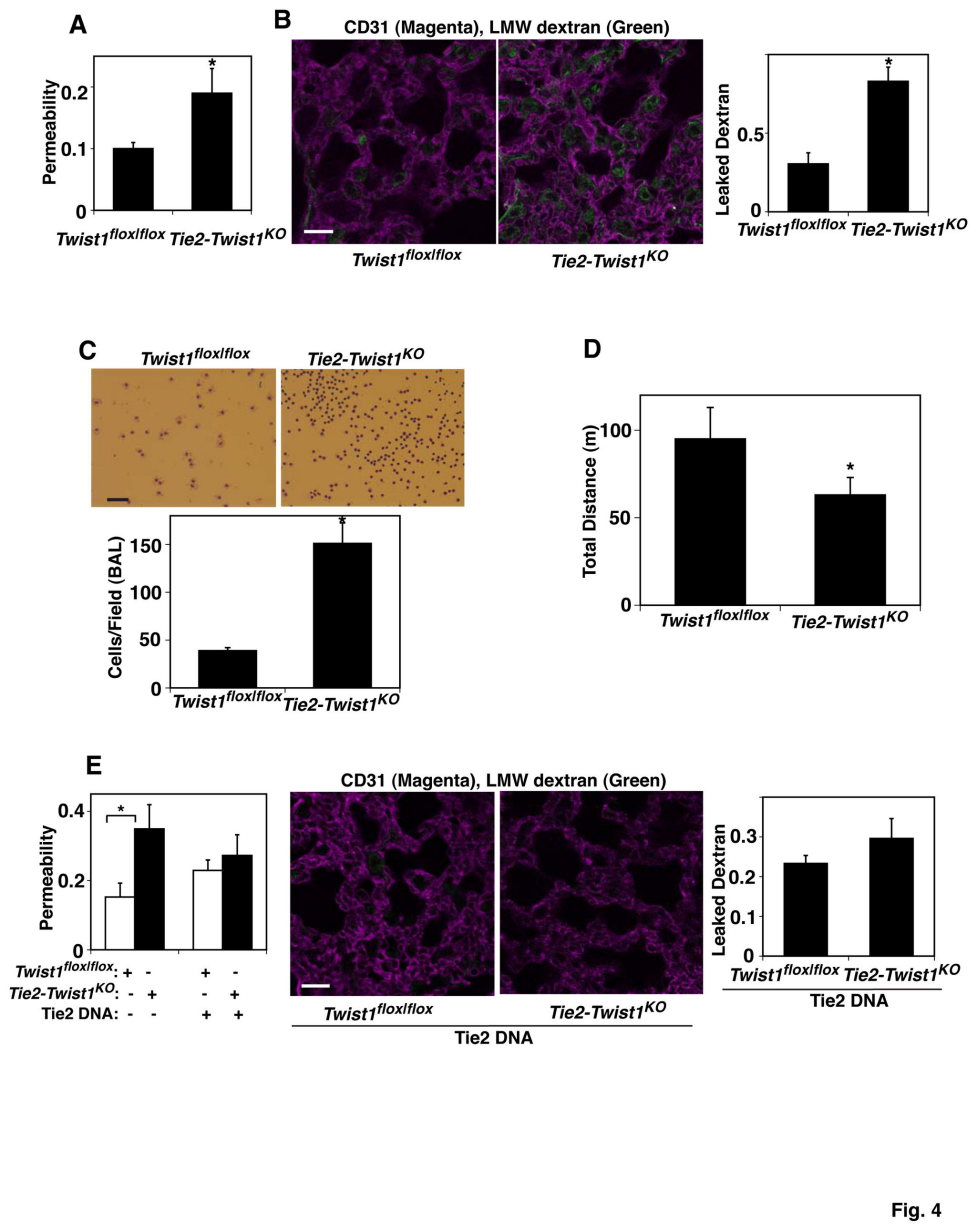
Twist1 regulates lung vascular permeability *in vivo*. **A**) Graph showing vascular permeability in the lungs of *Twist1* ^*flox|flox*^ or *Tie2-Twist1* ^KO^ mice. Vascular permeability is detected by Evans blue dye leakage in the lungs and extracted dye contents are quantified by measuring at 620 nm (n=9, *, p<0.05). **B**) Immunofluorescence micrographs showing LMW fluorescently labeled dextran leakage (green) and blood vessel formation (CD31 staining; magenta) in the lungs of *Twist1* ^*flox|flox*^ or *Tie2-Twist1* ^KO^ mice (bar, 20 µm). Graph showing the leaked dextran density normalized to vessel density in the lungs of *Twist1* ^*flox|flox*^ or *Tie2-Twist1* ^KO^ mice (n=7, *, p<0.05). **C**) Micrographs showing the immune cells in BAL fluid from the lungs of *Twist1* ^*flox|flox*^ or *Tie2-Twist1* ^KO^ mice detected by Wright-Giemsa staining (*Upper*, bar, 50 µm). Graph showing the immune cell count in BAL fluid from the lungs of *Twist1* ^*flox|flox*^ or *Tie2-Twist1* ^KO^ mice (*Lower*, n=8, *, p<0.05). **D**) Exercise capacity of *Twist1* ^*flox|flox*^ or *Tie2-Twist1* ^KO^ mice assessed by total running distance according to a predetermined protocol (n=15, *, p<0.05). **E**) Graph showing vascular permeability detected by Evans blue dye leakage in the lungs of *Twist1* ^*flox|flox*^ or *Tie2-Twist1* ^KO^ mice treated with Tie2 DNA (*left*; n=9, *, p<0.05). Immunofluorescence micrographs showing LMW fluorescently labeled dextran leakage (green) and blood vessel formation (CD31 staining; magenta) in the lungs of *Twist1* ^*flox|flox*^ or *Tie2-Twist1* ^KO^ mice treated with Tie2 DNA (*right*; bar, 20 µm). Graph showing the leaked dextran density normalized to vessel density in the lungs of *Twist1* ^*flox|flox*^ or *Tie2-Twist1* ^KO^ mice treated with Tie2 DNA (n=7). As a control, mice were treated with control DNA (vector only). All error bars are s.e.m.

To further determine whether changes in Twist1 expression regulate pulmonary vascular permeability *in vivo*, we measured vascular permeability in *Tie2-Twist1* ^KO^ mouse lungs by measuring leakage of Evans blue dye (Figure 4A) or fluorescently labeled low molecular weight (LMW) dextran (Figure 4B). The leakage of Evans blue dye into lung extravascular space was 1.8 times higher in *Tie2-Twist1* ^KO^ mouse lungs compared to that in *Twist1* ^*flox|flox*^ control mice (Figure 4A). The leakage of fluorescently labeled LMW dextran into lung alveolar spaces also increased by 2.5-fold in *Tie2-Twist1* ^KO^ mouse lungs compared to that in control *Twist1* ^*flox|flox*^ mice (Figure 4B). In addition, Twist1 knockdown in *Tie2-Twist1* ^KO^ mouse lungs increased the number of immune cells in bronchoalveolar lavage (BAL) fluid by approximately 3-fold, further indicating that vascular permeability is increased in *Tie2-Twist1* ^KO^ mouse lungs (Figure 4C). Exercise capacity as measured by the total distance mice were able to run using a rodent treadmill exercise protocol, was decreased by 25% in *Tie2-Twist1* ^KO^ mice compared to control *Twist1* ^*flox|flox*^ mice (Figure 4D), indicating that Twist1 plays a key role in physiological lung function. Consistent with the data from *in vitro* study, Tie2 overexpression using retro-orbital injection of cationic DNA [7,36,37], which increases Tie2 protein levels in the lungs (Figure S1A), partially restored lung vascular leakage in *Tie2-Twist1* ^KO^ mice (Figure 4E), confirming that Twist1-Tie2 signaling controls lung vascular barrier function *in vivo*.

### Twist1-Tie2 signaling mediates endotoxin-induced lung injury

**Figure 5:**
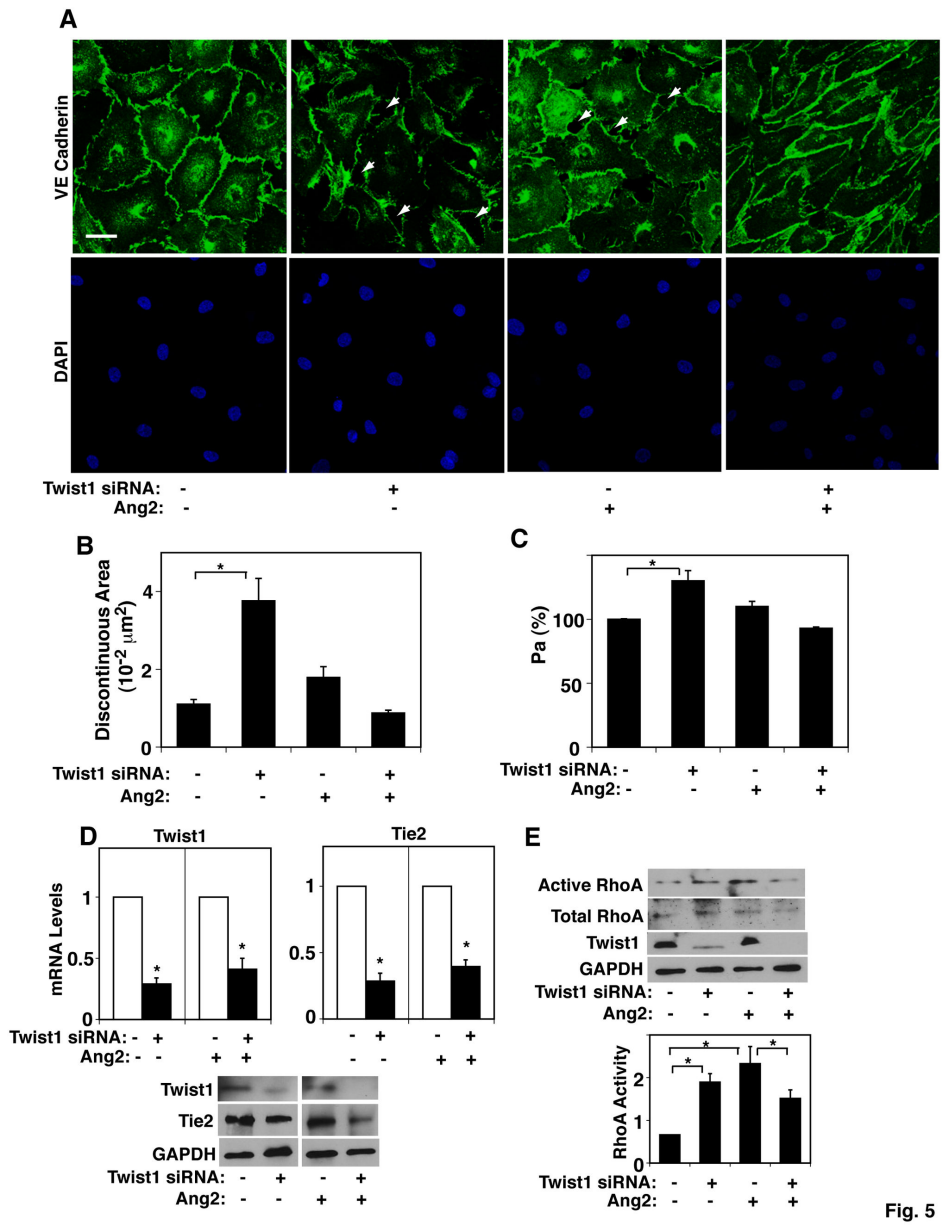
Twist1 knockdown fails to disrupt endothelial cell-cell junction integrity in L-HMVE cells under Ang2 treatment *in vitro*. **A**) Immunofluorescence micrographs showing cell-cell junction structure by VE-cadherin staining in L-HMVE cells treated with Twist1 siRNA (#1), Ang2, or both in combination (bar, 50 µm). DAPI staining shows the nucleus of each cell. Arrows show the region where cell-cell junctions are disrupted. As a control, cells were treated with siRNA duplex with an irrelevant sequence. **B**) Graph showing the quantitation of the total discontinuous area in L-HMVE cells treated with Twist1 siRNA (#1), Ang2, or both in combination in at least 10 fields (*, p<0.05). **C**) Graph showing endothelial permeability in L-HMVE cells treated with Twist1 siRNA (#1), Ang2, or both in combination (*, p<0.05). Permeability (Pa) values are expressed as percentage of control cells. **D**) Graphs showing Twist1 and Tie2 mRNA levels in L-HMVE cells treated with Twist1 siRNA (#1), Ang2, or both in combination (*top*, * p<0.01). Immunoblots showing Twist1, Tie2, and GAPDH protein levels in L-HMVE cells treated with Twist1 siRNA (#1), Ang2, or both in combination (bottom). **E**) Immunoblots showing levels of active RhoA in L-HMVE cells treated with Twist1 siRNA (#1), Ang2, or both in combination (top). Quantitative results (ratio of active RhoA to total RhoA) were normalized to control siRNA treated Ang2-untreated cells (*, p<0.05). All error bars are s.e.m.

Circulating serum Ang2 is elevated in humans with various pathological conditions such as ARDS, cancer and inflammation, in which vascular permeability is elevated [12,13,38–41]. Although Twist1 knockdown using siRNA transfection disrupted cell-cell junctional integrity in L-HMVE cells, when cells were treated with Ang2, Twist1 knockdown failed to disrupt cell-cell junctional integrity (Figure 5A). Quantitative results revealed that the discontinuous area was increased by 3-fold in Twist1 knockdown L-HMVE cells, which was restored to the levels of untreated control cells when treated with Ang2 (Figure 5B). Consistently, Twist1 knockdown failed to increase vascular permeability, measured using a transwell vascular permeability assay, under Ang2 treatment in L-HMVE cells (Figure 5C). When we measured the mRNA and protein levels of Twist1 and Tie2 in L-HMVE cells, they were 60-70% lower in Twist1-knocked down Ang2-treated L-HMVE cells, which were identical to untreated cells (Figure 5D), suggesting that Ang2 does not affect the Twist1 knockdown-induced decrease in Tie2 expression. We also examined whether Twist1 controls RhoA activity in Ang2-treated L-HMVE cells. When we examined RhoA activity using a rhotekin pull down assay, RhoA activity was lower by 30% in Twist1-knocked down Ang2-treated L-HMVE cells compared to the cells treated with Ang2 and control siRNA with irrelevant sequence (Figure 5E).

**Figure 6:**
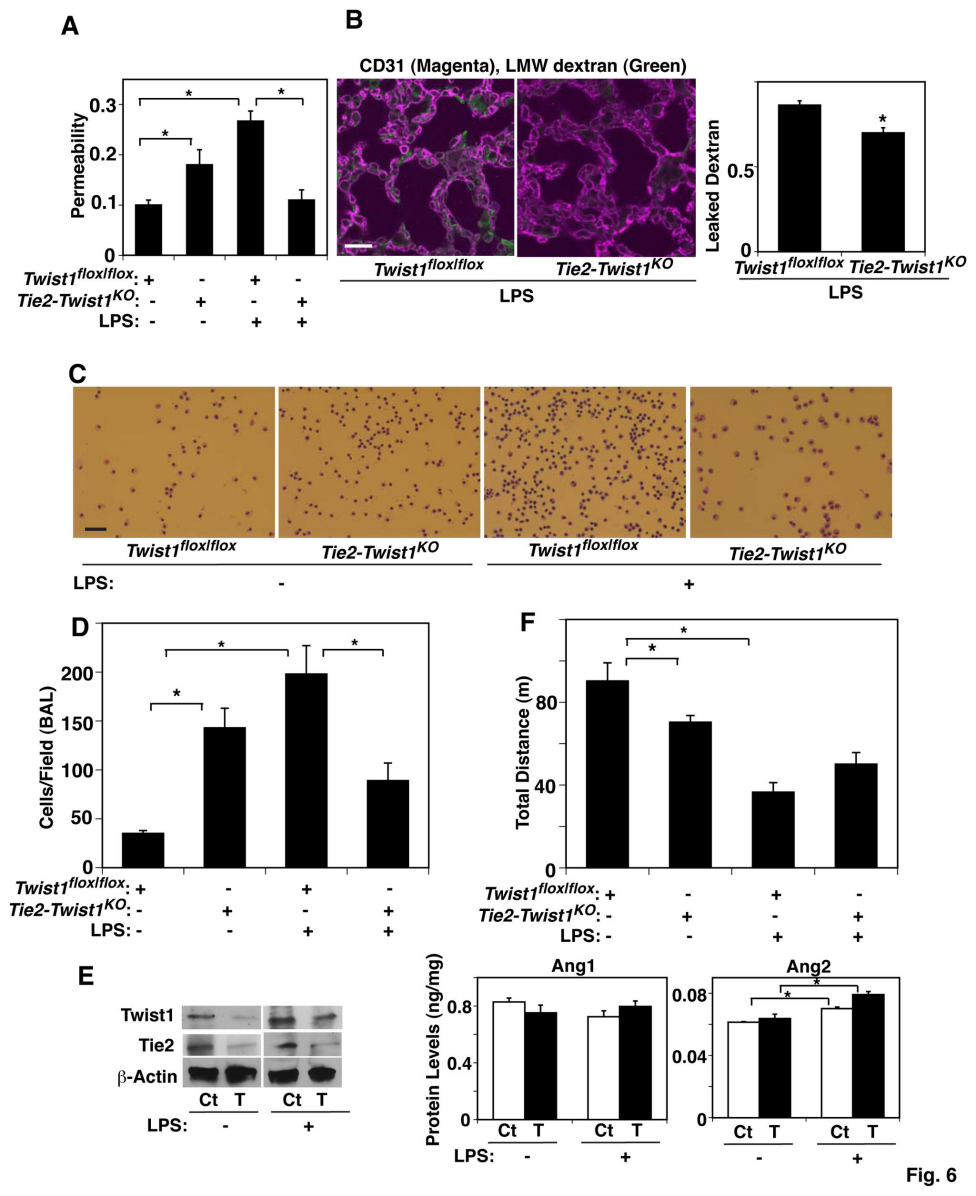
Twist1 mediates lung vascular permeability in endotoxin-induced lung injury *in vivo*. **A**) Vascular permeability detected by Evans blue dye leakage in the lungs of *Twist1* ^*flox|flox*^ or *Tie2-Twist1* ^KO^ mice treated with LPS for 1 day (n=10, *, p<0.05). **B**) Immunofluorescence micrographs showing LMW fluorescently labeled dextran leakage (green) and blood vessel formation (CD31 staining; magenta) in LPS-treated lungs of *Twist1* ^*flox|flox*^ or *Tie2-Twist1* ^KO^ mice (bar, 20 µm). Graph showing the leaked dextran density normalized to vessel density in the lungs of LPS-treated *Twist1* ^*flox|flox*^ or *Tie2-Twist1* ^KO^ mice (n=7, *, p<0.05). **C**) Micrographs showing the immune cells in BAL fluid from the lung of *Twist1* ^*flox|flox*^ or *Tie2-Twist1* ^KO^ mice treated with LPS for 1 day, detected by Wright-Giemsa staining (bar, 50 µm). **D**) Graph showing the immune cell count in BAL fluid from the lungs of *Twist1* ^*flox|flox*^ or *Tie2-Twist1* ^KO^ mice treated with LPS for 1 day (n=8, *, p<0.05). **E**) Immunoblots showing Twist1, Tie2, and β-actin protein levels in the lungs of *Twist1* ^*flox|flox*^ (Ct) or *Tie2-Twist1* ^KO^ mice (T) treated with LPS for 1 day (left). Graphs showing Ang1 and Ang2 protein levels in the lungs of *Twist1^flox|flox^* (Ct) or *Tie2-Twist1* ^KO^ mice (T) treated with LPS for 1 day (*right*, n=8, * p<0.05). **F**) Exercise capacity of *Twist1* ^*flox|flox*^ or *Tie2-Twist1* ^KO^ mice treated with LPS for 1 day assessed by total running distance according to a predetermined protocol (n=15, *, p<0.05). All error bars are s.e.m.

To determine whether Twist1-Tie2 signaling mediates lung vascular permeability in sepsis-induced lung injury *in vivo*, we exposed whole lung of living adult mice to the endotoxin, lipopolysaccharide (LPS), which induces the development of pulmonary edema and ARDS in humans with sepsis [3,42]. Systemic LPS treatment is a widely accepted physiological animal model for sepsis-induced ARDS [3,7,31,43]. Consistent with previous reports [3,7], LPS treatment for 24 hours in control *Twist1* ^*flox|flox*^ mice increased lung vascular permeability by 2.5-fold compared to untreated control mice when measured using Evans blue dye leakage (Figure 6A). LPS-induced increase in lung vascular permeability was significantly suppressed in *Tie2-Twist1* ^KO^ mice, in which Tie2 expression in lungs was decreased (Figure 3B–E), compared to control *Twist1* ^*flox|flox*^ mice (Figure 6A). We also confirmed the effects of LPS treatment in *Tie2-Twist1* ^KO^ mice using fluorescent-labeled LMW dextran leakage, showing that increased dextran leakage into alveolar spaces was 15% lower in *Tie2-Twist1* ^KO^ mice compared to *Twist1* ^*flox|flox*^ mice (Figure 6B). Consistently, the number of immune cells in the BAL fluid increased by 4.5-fold in the LPS-treated control *Twist1* ^*flox|flox*^ mice, while this effect was attenuated in the *Tie2-Twist1* ^KO^ mice (Figure 6C, D). These results suggest that down-regulated Twist1-Tie2 signaling suppresses endotoxin-induced vascular leakage in mouse lungs. Consistent with *in vitro* results (Figure 5D), the mRNA and protein levels of Twist1 and Tie2 in the lungs were 30-70% lower in *Tie2-Twist1* ^KO^ mice regardless of LPS treatment (Figure 6E, Figure S2). Importantly, LPS treatment significantly increased the protein levels of Ang2 in the lungs, while it did not change Ang1 levels (Figure 6E), suggesting that downregulation of Twist1-Tie2 signaling prevents the LPS-induced increase in lung vascular permeability by suppressing the effects of its ligand, Ang2, on endothelial cell-cell junctional integrity. Although basal exercise capacity measured using a rodent treadmill exercise protocol was significantly lower in LPS-treated *Twist1* ^*flox|flox*^ mice compared to untreated mice, when evaluated 1 week after the LPS treatment, the running ability was partially reversed in LPS-treated*Twist1* ^*flox|flox*^ mice (Figure 6F). These findings suggest that down-regulation of Twist1-Tie2 signaling attenuates the endotoxin-induced increase in vascular permeability and restores lung function.

## Discussion

**Figure 7:**
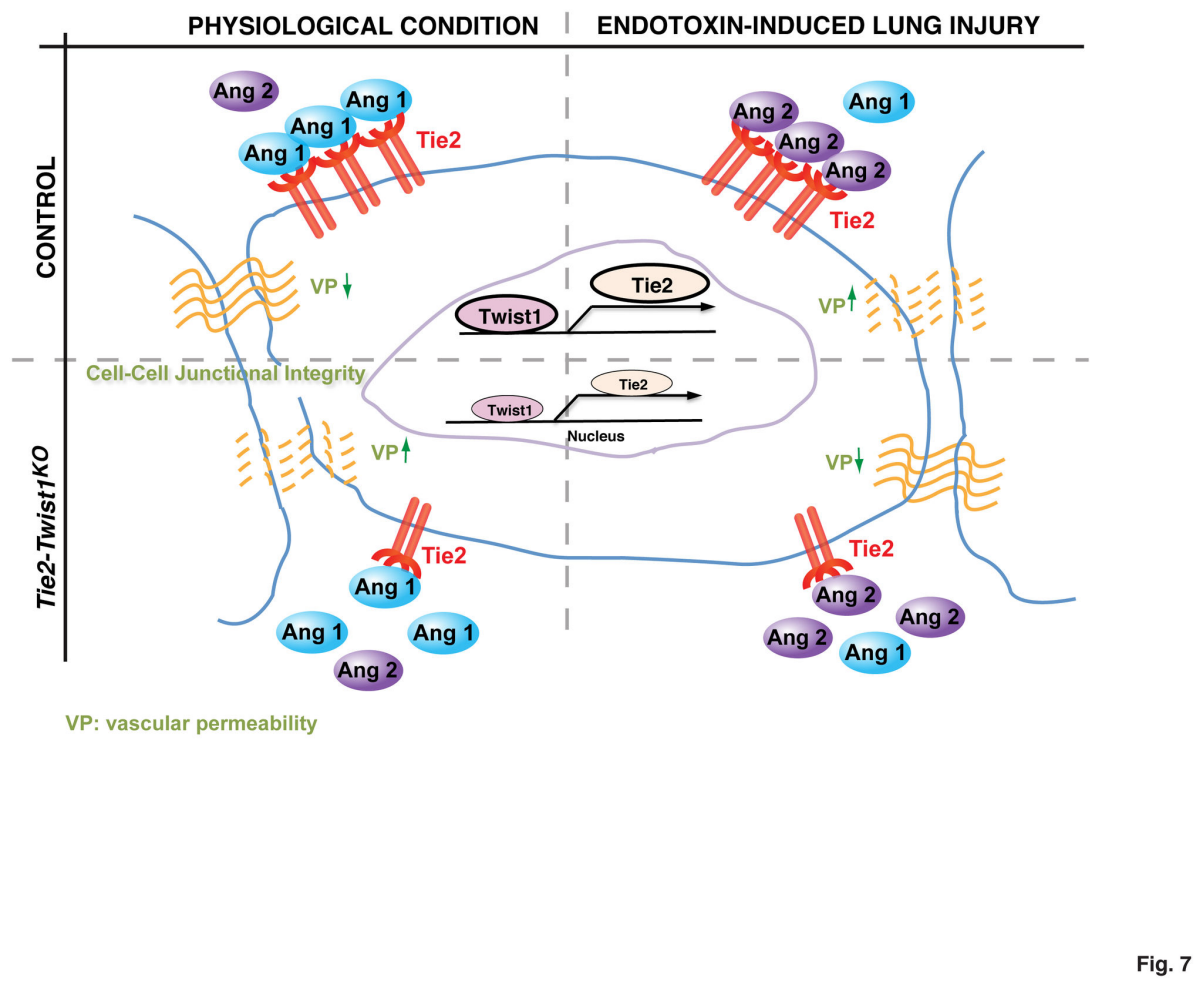
Twist1 regulates lung vascular barrier function through the Ang-Tie2 pathway. Vascular permeability in the lung can be controlled by Twist1-Tie2 signaling depending on the Ang1/Ang2 ratio. In physiological conditions where the ratio of Ang1 and Ang2 is in favor of Ang1, knockdown of Twist1, which decreases the expression of Tie2, increases vascular permeability in the lung. However, during a pathological condition such as endotoxin-induced lung injury, in which Ang2 is upregulated [12,13,38], knockdown of Twist1 fails to increase vascular permeability, rather reverses the increase of vascular leakage in the lungs.

Tightly regulated vascular permeability is critical to maintain lung function, while deregulated vascular permeability contributes to the pathogenesis of acute lung injury and ARDS [3,31,32]. Here we show that Twist1 controls lung vascular permeability by altering Tie2 expression. In physiological conditions where the ratio of Ang1 and Ang2 is in favor of Ang1, knockdown of Twist1 decreases Tie2 expression and hence increases lung vascular permeability in cultured L-HMVE cells *in vitro* and in adult mouse lung *in vivo*. However, in pathological conditions in which Ang2 is upregulated [12,13,38], downregulation of Twist1-Tie2 signaling prevents the increase of vascular leakage in the lungs by attenuating the vessel-destabilizing effects of Ang2 (Figure 7). These findings suggest that Twist1- Tie2 signaling in combination with the signatures of Angs regulates lung vascular permeability in a context-dependent way. Since the detrimental effects of endotoxin on pulmonary vascular permeability can be prevented by suppressing Twist1 expression, targeting the Twist1-Tie2 pathway could potentially lead to the development of new approaches for sepsis-induced ARDS and other diseases with abnormal vascular permeability in the future.

Our results revealed that Tie2 overexpression partly, but not completely, restored the Twist1 knockdown-induced lung vascular leakage *in vitro* and *in vivo*. Given that Twist1 has a b-HLH sequence, which binds to an E-box sequence [18,29], and that E-box also exists in the promoter region of VEGFR2 [44] and VE-cadherin [45], which also control cell-cell junctional integrity and vascular permeability, Twist1 may control cell-cell junctional integrity and vascular permeability by modulating the expression of these molecules as well. In fact, Twist1 knockdown in L-HMVE cells decreased the expression of VEGFR2 at both mRNA and protein levels (Figure S1B). Thus, Twist1 may alter lung vascular permeability by changing the expression of VEGFR2 as well. Our finding showing that Twist1 as a regulator of Tie2 expression could be quite important as a general mechanism of endothelial Tie2 expression. Tie2 mediates physiological angiogenesis [46–48] and deregulation of this mechanism contributes to various pathological conditions such as bronchopulmonary dysplasia (BPD) [14] and tumor angiogenesis [49]. Since Twist1 positively regulates tumor angiogenesis [50,51], Twist1-Tie2 signaling may be involved in the mechanisms of tumor-angiogenesis as well, in which vascular permeability is increased. Further investigation of the roles of the Twist1-Tie2 pathway on physiological and pathological angiogenesis as well as vascular barrier function will likely expand our scientific knowledge and lead to the development of new therapeutic strategies for angiogenesis-related diseases.

It has been known that Ang1 and Ang2 bind to their common receptor Tie2, antagonize each other and control blood vessel maturation and stabilization [52]; Ang1 stabilizes blood vessel formation [3,52–54], whereas Ang2 destabilizes the blood vessel structure and increases vascular permeability in lung injury [12,13,55–57], tumors [39–41] and lung fibrosis [58,59]. Our findings suggest that downregulation of Twist1 expression inhibits the Ang1-induced vascular stabilization in physiological conditions, whereas it prevents the Ang2-induced vascular destabilization in pathological conditions (i.e., endotoxin-induced lung injury) by decreasing the expression of its receptor Tie2. This is consistent with our previous report showing that Tie2 expression controlled by LRP5 and the balance of Ang1 and Ang2 coordinately regulate lung vascular development and contribute to the pathogenesis of BPD, in which lung vascular permeability is elevated [14]. It has been reported that Twist1 levels are higher in tumors and fibrotic tissues [24–28,50,60,61], in which Ang2 levels are high and vascular permeability is elevated. Therefore, in addition to manipulating the levels of Angs, modulating Tie2 expression through Twist1 could be a good therapeutic strategy for these diseases as well.

VEGF is a well-known vascular permeability factor [4,62]. Importantly, when the mouse paw was treated with Ang2 and VEGF at the same time, the effects were additive, which suggests that these two factors act independently [63,64]. However, it has also been reported that Ang1 has a protective function against increased brain barrier permeability caused by overexpression of VEGF [65]. In addition, Ang2 expression levels are regulated by VEGF [66] and VEGF-VEGFR2 signaling is known to interact with the Tie2/Angs system to modulate vascular functions [49,67,68]. Thus, Angs and VEGF control vascular permeability both independently and cooperatively, and play important roles in angiogenesis, vessel maturation and inflammation [64].

In addition to soluble angiogenic factors, cell-generated mechanical forces and adhesive properties to the ECM are also known to influence angiogenesis and organ morphogenesis [69,70]. Changes in ECM mechanics (stiffness) control VEGF-induced angiogenesis [34] and lung vascular permeability [7]. In fact, endotoxin-treated lungs [7], tumor tissues [71,72] and fibrotic lungs [73,74], in which microvessels are hyperpermeable, are accompanied by increased ECM stiffness. Since Twist expression is induced by mechanical forces (i.e., physical compression) in the Drosophila embryo [75,76], in addition to chemical regulators, physical changes in ECM mechanics might regulate lung vascular permeability via the Twist1-Tie2 pathway.

In summary, we have demonstrated that Twist1 regulates Tie2 expression and modulates lung vascular permeability in an Angs-dependent way. Since inhibition of this pathway prevents LPS-induced vascular leakage in the lung, the Twist1/Tie2 system could represent a novel therapeutic target for ARDS as well as other diseases caused by abnormal vascular permeability.

## Materials and Methods

### Materials

Anti -CD31 and –VE-cadherin monoclonal antibodies were from Transduction Laboratories (Lexington, KY). Anti-GAPDH monoclonal antibody was from Chemicon (Temecula, CA). Anti-β actin monoclonal antibody was from Sigma. Anti–Twist1 monoclonal and -RhoA polyclonal antibodies were from Abcam. Anti–Twist1 polyclonal antibody was from Santa Cruz Biotechnology (Dallas, TX). Anti-Tie2 monoclonal antibody and anti-phosphotyrosine (4G10) were from Upstate (Lake Placid, NY). Anti-VEGFR2 polyclonal antibody was from Cell Signaling (Danvers, MA). LPS was from Sigma. L-HMVE cells and HUVE cells (Lonza, Walkersville, MD) were cultured as described before [3,14].

### Plasmid construction and gene knockdown

The full length Tie2 plasmid (pSPORT Tie2) was from Open Biosystems (Huntsville, AL) and transient transfection was performed using Superfect reagent (QIAGEN, Valencia, CA) according to the manufacturer’s directions [34]. As a control, plasmid with vector only was used. Gene knockdown was performed using the RNA interference technique [34]. siRNAs for human Twist1 #1 was 5’- UUGAGGGUCUGAAUCUUGCUCAGCU -3’ and 5’- AGCUGAGCAAGAUUCAGACCCUCAA -3’. #2 was 5’-ACUCCAAGAUGGCAA GCUG-3’ and 5’- CAGCUUGCCAUCUUGGAGU-3’ [77]. As a control, siRNA duplex with an irrelevant sequence (QIAGEN) was used.

### Biochemical Methods

Rho activity assay was performed and quantified using the Rho activation assay kit based on rhotekin pull-down assay according to the manufacturer’s instruction (Cytoskeleton, Denver, CO) [78]. The ratio of rhotekin-bound RhoA and RhoA in the total cell lysate was analyzed using NIH ImageJ software [78]. The levels of Ang1 and Ang2 in the mouse lung homogenate were measured by ELISA (MyBioSource, San Diego, CA).

### Molecular biological methods

Quantitative reverse transcription (qRT)-PCR was performed with the Quantitect SYBR, Green RT-PCR kit (Agilent) using the ABI 7300 real time PCR system (Applied Biosystems, Foster City, CA). β2 microglobulin or cyclophilin controlled for overall cDNA content. The primers used for human and mouse Tie2, mouse Ang1 and Ang2, human VEGFR2, human β2 microglobulin and mouse cyclophilin were previously described [14,34]. The primers for human Twist1 were 5’-GTCCGCAGTCTTACGAGGAG-3’, 5’- GCTTGAGGGTCTGAATCTTGCT-3’, mouse Twist1 were 5’-GGACAAGCTGAGCAAGATTCA-3’, 5’- CGGAGAAGGCGTAGCTGAG-3’. For ChIP assay, DNA from L-HMVE cells transfected with control or Twist1 siRNA #1 was immunoprecipitated with the Twist1 antibody or control immunoglobulin (Jackson Immuno Research), according to the manufacturer instructions (Active Motif, Carlsbad, CA) [34]. The Twist1-binding region was amplified using primers, 5’-TTGCTTTTCAGGTTGTATTTTC-3’ and 5’-agaataacaagccctccacc-3’.

### Cell analysis methods

L-HMVE cell monolayer junction formation was analyzed using immunohistochemistry with VE-cadherin antibody staining [3,7,34]. L-HMVE cells were cultured for 12 h and immunostaining was performed and analyzed using confocal Leica SP2 microscope [34]. Discontinuous area was calculated using ImageJ software in ten random fields in three independent experiments [7]. L-HMVE cell monolayer permeability was determined with the use of FITC-labeled bovine serum albumin (Sigma) as described previously [3]. FITC-albumin (final concentration 1 mg/ml) was added to the luminal chamber for 6 h, and samples were taken from both the luminal and abluminal chamber for fluorometric analysis. Where indicated, vehicle or Ang2 (30 ng/ml, R&D systems) was added to the luminal chamber with FITC-labeled bovine serum albumin. Fluorescence readings were converted with the use of a standard curve to albumin concentration. These concentrations were used to determine the permeability coefficient of albumin (Pa) as described [3].

### In Vivo Pulmonary Permeability Assay

The *in vivo* animal study was carried out in strict accordance with the recommendations in the Guide for the Care and Use of Laboratory Animals of the National Institutes of Health. The protocol was reviewed and approved by the Animal Care and Use Committee of Boston Children’s Hospital (Protocol Number: 10-11-1818R). Mice with Tie2-specific knockdown of Twist1 (*Tie2-Twist1* ^*KO*^) were generated by cross-breeding Tie2-Cre expressing C57BL/6J mice (Jackson Laboratory stock #004128) with Twist1 floxed mice (*Twist1* ^*flox|flox*^) [79]. Mice (6–8 weeks old) were treated with LPS (2.5 mg/kg, intraperitoneally) and lung permeability was assessed 24 hours after injection [3,7]. For gene overexpression, delivery of DNA into mice was performed using retro-orbital injection of the mixture with Exgen (Fermentas) according to the manufacturer’s instructions [7,36,37]. Gene overexpression in the lung (2 days later) was confirmed by measuring protein levels using immunoblotting. The lung permeability was measured using Evans blue dye or LMW fluorescently labeled dextran (MW 4000, sigma) leakage [3,7]. Evans blue dye was extracted from the lung by incubation with formamide (70 °C for 24 h) and the absorbance of extracted dye was measured at 620 nm. Dextran leakage was quantified using a macro designed for NIH’s ImageJ software that counts colored pixels between thresholds selected to minimize background, yielding a percentage of total image area, which was then normalized to vessel density, with each parameter analyzed independently.

For TEM, small pieces (1-2 mm cubes) of lung tissue were fixed with 2.5% Glutaraldehyde and 2% Paraformaldehyde in 0.1 M sodium cacodylate buffer (pH 7.4) for at least 2 h at room temperature, washed in 0.1M cacodylate buffer and postfixed with 1% Osmium tetroxide (OsO4)/1.5% Potassiumferrocyanide (KFeCN6) for 1 h, washed in water and incubated in 1% aqueous uranyl acetate for 1 h followed by washes in water and subsequent dehydration in grades of alcohol. The samples were then put in propylene oxide for 1 hr and infiltrated ON in a 1:1 mixture of propylene oxide and TAAB Epon (Marivac Canada Inc, St.-Laurent, Canada). The samples were then embedded in TAAB Epon and polymerized at 60 °C for 48 hrs. Ultrathin sections (about 60 nm) were cut on a Reichert Ultracut-S microtome, picked up on to copper grids stained with lead citrate and examined in a TecnaiG² Spirit BioTWIN.

BAL was performed by instilling 0.9% NaCl in two separate 0.5 ml aliquots. The fluid was recovered by gentle suction and placed on ice for immediate processing. An aliquot of the BAL fluid was processed immediately for differential cell counts by performing cytospin preparations and staining with modified Wright-Giemsa stain (Diff-Quik; American Scientific Products, McGaw Park, IL) [7].

### Laser-Capture Microdissection of Lung Vessels

Lung vessels were microdissected with laser capture in unfixed cross sections of lungs from *Tie2-Twist1* ^*KO*^ and *Twist1* ^*flox|flox*^ mice, as described previously [80–82]. Lungs from *Tie2-Twist1* ^*KO*^ and *Twist1* ^*flox|flox*^ mice were collected after injecting fluorescein-conjugated Concanavalin A into the retro-orbital vein to stain blood vessels [7,34]. Lungs were embedded in OCT, cut into 8-µm sections, and collected on RNase-free polyethylene naphthalate membrane slides (Leica). Blood vessels were microdissected on a LCM system (LMD-6000; Leica). RNA was extracted from microdissected tissues (RNeasy Micro Kit; Qiagen), and real-time PCR was performed.

### Isolation of CD31-positive cells from mouse lungs

The lung was minced into small pieces and digested in a solution of 1 mg/ml collagenase (Sigma, St. Louis, MO) and 2.4 U/ml dispase solution (Collaborative Biomedical Products, Bedford, MA) for 40 min at 37 °C. Single-cell suspensions were incubated with anti-CD31 antibody-conjugated microbeads (Miltenyl Biotec, Auburn, CA, USA) on ice and the CD31-positive population was isolated according to the manufacturer’s instruction.

### Exercise Capacity

Mice were run according to a predetermined protocol and we assessed the ability of untrained mice to run for distance [83,84]. The animals were initially acclimated to the treadmill environment for 30 min. For warm-up and for further familiarization with treadmill running, the mice were required to run at a relatively easy pace of 10 m/min for 30 min. Then the speed of the treadmill was increased to 20 m/min, and we recorded the exercise duration and distance the mice could run until exhaustion. Exhaustion was defined operationally as the time at which the mouse was unable, or refused, to maintain its running speed despite encouragement by mild electrical stimulation.

### Statistical analysis

All phenotypical analysis was performed by masked observers unaware of the identity of experimental groups. All statistical data was analyzed using GraphPad Prism V 5.0. Error bars (SEM) and *p* values were determined from the results at least three or more independent experiments. The ANOVA with post-hoc student T test was used for analysis of statistical significance.

## Supporting Information

Figure S1 **Tie2 overexpression in mouse lungs and VEGFR2 expression in Twist1 knockdown L-HMVE cells.** **A**) Immunoblots showing Tie2 and GAPDH protein levels in mouse lungs treated with Tie2 DNA. As a control, mouse was treated with control DNA (vector only). **B**) Immunoblots showing VEGFR2, Twist1 and GAPDH protein levels in L-HMVE cells treated with Twist1 siRNA #1 (left). Graph showing Twist1 and VEGFR2 mRNA levels in L-HMVE cells treated with Twist1 siRNA #1 (* p<0.01). As a control, cells were treated with siRNA duplex with an irrelevant sequence. Error bars represent s.e.m. of at least three independent experiments.
(TIF)

Figure S2 **Expression of Twist1 and Tie2 in LPS-treated lungs *in vivo*.** Graphs showing Twist1 and Tie2 mRNA levels in the lungs of *Twist1^flox|flox^* (Ct) or *Tie2-Twist1* ^KO^ (T) mice treated with LPS (n=8, * p<0.05). Error bars are s.e.m.
(TIF)
