# Supplementary figures and images for: Twist1 Controls Lung Vascular Permeability and Endotoxin-Induced Pulmonary Edema by Altering Tie2 Expression
Source: PLoS One. 2013 Sep 2;8(9):e73407. doi: 10.1371/journal.pone.0073407 (PMC3759405; doi:10.1371/journal.pone.0073407)

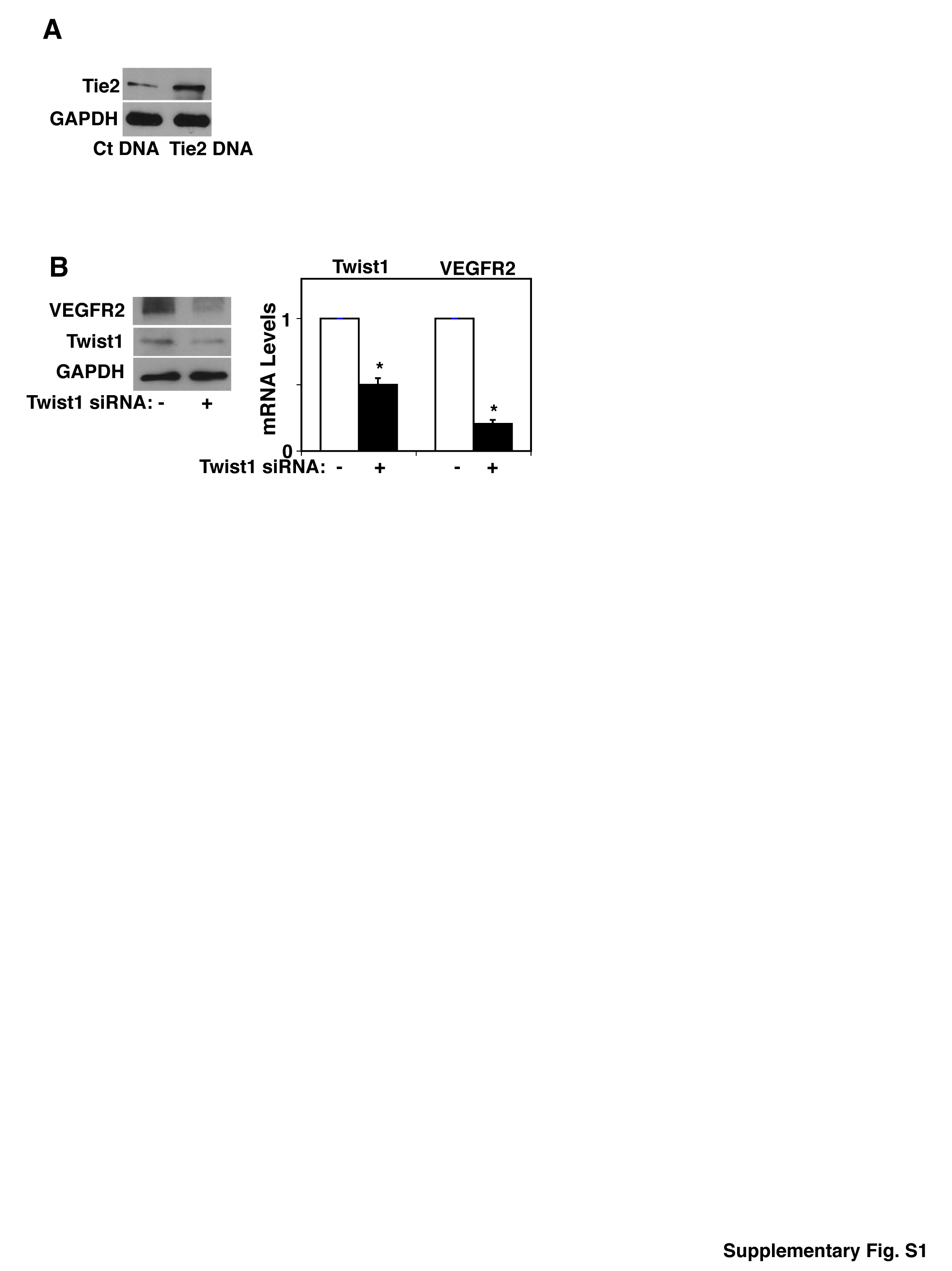

Supplement: Figure S1 — Tie2 overexpression in mouse lungs and VEGFR2 expression in Twist1 knockdown L-HMVE cells. A) Immunoblots showing Tie2 and GAPDH protein levels in mouse lungs treated with Tie2 DNA. As a control, mouse was treated with control DNA (vector only). B) Immunoblots showing VEGFR2, Twist1 and GAPDH protein levels in L-HMVE cells treated with Twist1 siRNA #1 (left). Graph showing Twist1 and VEGFR2 mRNA levels in L-HMVE cells treated with Twist1 siRNA #1 (* p<0.01). As a control, cells were treated with siRNA duplex with an irrelevant sequence. Error bars represent s.e.m. of at least three independent experiments. (TIF) [file pone.0073407.s001.tif]

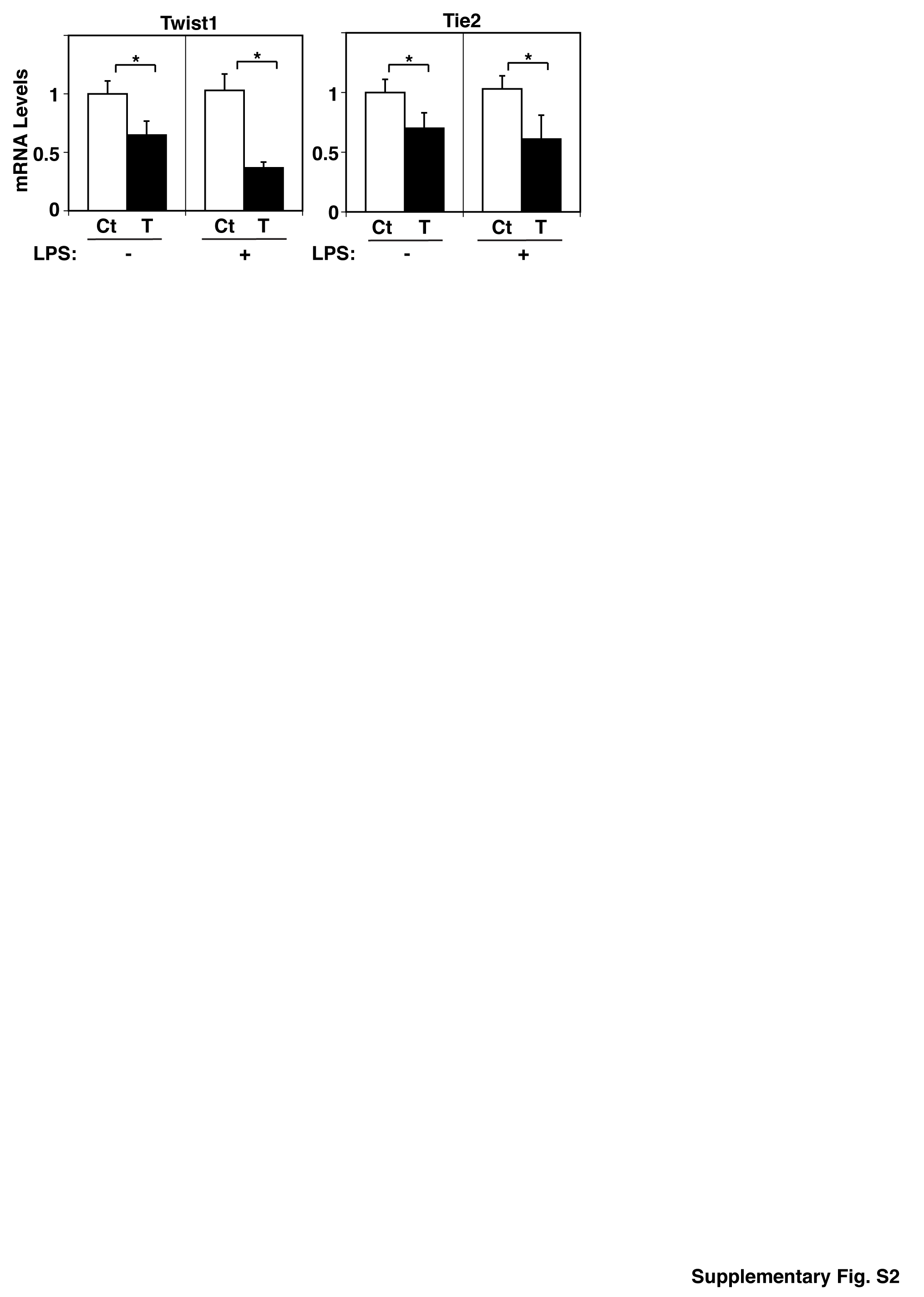

Supplement: Figure S2 — Expression of Twist1 and Tie2 in LPS-treated lungs in vivo. Graphs showing Twist1 and Tie2 mRNA levels in the lungs of Twist1flox|flox (Ct) or Tie2-Twist1 KO (T) mice treated with LPS (n=8, * p<0.05). Error bars are s.e.m. (TIF) [file pone.0073407.s002.tif]
